# Early reoperation following pancreaticoduodenectomy: impact on morbidity, mortality, and long-term survival

**DOI:** 10.1186/s12957-019-1569-9

**Published:** 2019-01-31

**Authors:** Yonatan Lessing, Niv Pencovich, Nadav Nevo, Nir Lubezky, Yaacov Goykhman, Richard Nakache, Guy Lahat, Joseph M. Klausner, Ido Nachmany

**Affiliations:** Department of General Surgery B, Division of General Surgery, Tel-Aviv Sourasky, Medical Center, Affiliated with the Sackler Faculty of Medicine, Tel Aviv University, 6 Weizmann St., 64239 Tel-Aviv, Israel

**Keywords:** Pancreas, Whipple, Surgery, Complication

## Abstract

**Background:**

Reoperation following PD is a surrogate marker for a complex post-operative course and may lead to devastating consequences. We evaluate the indications for early reoperation following PD and analyze its effect on short- and long-term outcome.

**Methods:**

Four hundred and thirty-three patients that underwent PD between August 2006 and June 2016 were retrospectively analyzed.

**Results:**

Forty-eight patients (11%; ROp group) underwent 60 reoperations within 60 days from PD. Forty-two patients underwent 1 reoperation, and 6 had up to 6 reoperations. The average time to first reoperation was 10.1 ± 13.4 days. The most common indications were anastomotic leaks (22 operations in 18 patients; 37.5% of ROp), followed by post-pancreatectomy hemorrhage (PPH) (14 reoperations in 12 patients; 25%), and wound complications in 10 (20.8%). Patients with cholangiocarcinoma had the highest reoperation rate (25%) followed by ductal adenocarcinoma (12.3%). Reoperation was associated with increased length of hospital stay and a high post-operative mortality of 18.7%, compared to 2.6% for the non-reoperated group. For those who survived the post-operative period, the overall and disease-free survival were not affected by reoperation.

**Conclusions:**

Early reoperations following PD carries a dramatically increased mortality rate, but has no impact on long-term survival.

## Introduction

Pancreaticoduodenectomy (PD) is among the most complex and demanding operations. In spite of major advancements in surgical experience and perioperative care, the morbidity following this operation remains high relative to other abdominal operations, and the mortality is not trivial [1–4]. Since most patients are operated on for malignancy, one important implication of complex and prolonged post-operative course is failure to reach adjuvant treatment [5, 6]. Performing PD on older and sicker patients, the addition of vascular resections and use of neoadjuvant chemotherapy, all may play a role in the complexity of post-PD course. Frequent life-threatening complications include anastomotic leak, post-operative pancreatic fistula (POPF), early and delayed post-pancreatectomy hemorrhage (PPH), and abdominal sepsis and intra-abdominal abscess formation [1–3, 7]. Progress in gastrointestinal endoscopy and invasive radiology allows for non-operative management in most of these cases [8–10]. Nevertheless, surgery is still required when these measures fail or when a rapid clinical deterioration dictates a prompt or definitive solution. Previous reports showed that reoperation following PD is associated with higher rates of in-hospital mortality, increased length of hospital stay (LOS), and development of other post-operative complications including delayed gastric emptying (DGE), POPF, and PPH as well as systemic complications. However, the impact of reoperation on long-term survival is still unclear [11–13]. In this study, we evaluated the indications for early reoperation following PD and analyzed its effect on short outcome, as well as long-term outcomes of those operated for cancer.

## Materials and methods

### Patients

Included in this study are patients that underwent PD between August 2006 and June 2016. All operations were performed with a curative intent. The data were retrospectively retrieved from a prospectively collected database. Patients’ demographics and preoperative comorbidities including hypertension (HTN), ischemic heart disease (IHD), diabetes mellitus (DM), and chronic obstructive pulmonary disease (COPD) were evaluated. Additional preoperative data included standard lab tests, as well as serum levels of carcinoembryonic antigen (CEA) and CA19-9. The post-operative course including type and timing of complications, management including the usage of interventional radiology, and indications for reoperation, LOS, pathology report, adjuvant therapy data, and disease-free and overall survival were also evaluated. This study was approved by the Tel-Aviv Sourasky Medical Center Institutional Review Board.

### Statistical analysis

Statistical analysis was performed using the IBM SPSS statistics data editor. Continuous data is expressed as median values with the corresponding standard deviation. Student’s *t* test was used for continues data, and Fischer test and chi-square test were used for categorical data. Cumulative survival curves were plotted using the Kaplan-Meier method and statistically compared using the log-rank test.

## Results

Between August 2006 and June 2016, 433 patients underwent PD. Indications included pancreatic ductal adenocarcinoma (PDAC) in 226, ampullary carcinoma in 53, intra-ductal papillary mucinous neoplasm (IPMN) in 43, pancreatic neuroendocrine tumor (NET) in 33, distal cholangiocarcinoma (CCA) in 20, periampullary/duodenal carcinoma in 16, and solid-pseudopapillary neoplasm (SPN) in 4 patients. Eighteen patients were operated on for other pathologies, including mucinous cystic neoplasms, metastatic lesions, and soft tissue sarcomas. Twelve patients underwent PD for focal inflammatory lesions on imaging, without a preoperative histological proof of malignancy but a negative serology for IgG4-related autoimmune pancreatitis. Overall, eight patients (1.8%) underwent PD for presumed malignancy with an eventual benign pathology.

In 48 patients (11.0%) post-operative complications required at least one reoperation within 60 days of PD. The average time to first reoperation was 10.1 ± 13.4 days. Forty-two patients underwent 1, 4 underwent 2, and the remaining 2 patients underwent 4 and 6 reoperations (a total of 60 operations; 48 patients). The most common indication was an anastomotic leak (22 reoperations in 18 patients, 4.1% of all PD patients), followed by PPH (14 reoperations in 12 patients, 2.77% of all patients), and wound dehiscence (10 patients, 2.3% of all patients) (Table 1). Additional 14 reoperations were performed for miscellaneous reasons, as described below.

**Table 1:** Indications for reoperations

Indication for reoperation	No. of reoperations, total 60	No. of patients undergoing a reoperation	Days from primary surgery mean (range)
Anastomotic leak	22 (36.6%)	18	14.7 (1–28)
GJ	11 (50%)	9	12 (2–28)
PJ	9 (40.9%)	9	9 (2–18)
HJ	2 (9.1%)	2	1 (0)
Early bleeding	12 (20%)	10	2.8 (0–6)
Delayed bleeding (GDA)	2 (3.33%)	2	8 (7–9)
Bowel evisceration	10 (16.6%)	10	18.4 (5–14)
Uncontrolled sepsis	8 (13.3%)	8	6.4 (1–16)
Other	6 (10%)	5	14.1 (1–60)

*GJ* gastro-jejunostomy, *PJ* pancreatico-jejunostomy, *HJ* hepatico-jejunostomy, *GDA* gastroduodenal artery

The most common anastomotic leak leading to reoperation was gastro-jejunostomy (GJ) (11 reoperations in 9 patients; 2.2% of all patients), followed by pancreatico-jejunostomy (PJ) (9 patients; 2.2% of all patients), and hepatico-jejunostomy (HJ) (2 patients; 0.5%) (Table 1). Two patients underwent separate reoperations for both GJ and PJ leaks (Table 1). All GJ anastomoses were hand sewn, two layered, with an inner continuous absorbable suture, followed by interrupted non-absorbable, second layer. Of note, intra-abdominal drains posterior and anterior to the HJ and the PJ anastomoses, and reaching the GJ were placed routinely in all patients. The average time from PD to reoperation for anastomotic leak was 14.7± 13.7 days in GJ leak and 9.5 ± 12.8 days in PJ leak. The two patients that required reoperation due to HJ leak were operated on the first post-operative day (Table 1).

Twelve patients (2.77% of all PDs) underwent 14 operations due to uncontrolled bleeding. In 10 (12 operations), the bleeding was considered “early”—the mean time interval from PD to reoperation was 2.8 ± 2.4 days. In four of them, the source of bleeding was a branch of the superior mesenteric artery. Altogether, these patients required six operations. In one patient, the bleeding was from a branch of the superior mesenteric vein. In four, the bleeding was attributed to other sources including the abdominal wall, the gastric lumen (this patient was operated following a failure of endoscopic treatment), the short gastric vessels, and the pancreatic stump. In one patient, no bleeding source was found (Table 1). Two patients had delayed bleeding (on POD 7 and 9) due to rapture of a pseudo-aneurysm of the gastroduodenal artery (GDA) stump, secondary to POPF.

Eight patients (1.8% of all patients) underwent abdominal lavage and drainage due to uncontrolled sepsis. The mean time-interval to reoperation was 8.84.1 ± days. In all of them, no visible leak or clear source of sepsis were found (Table 1). Ten patients (2.3%) were operated due to wound dehiscence with bowel evisceration. The mean time to surgery was 16.1 ± 15.6 days (Table 1). For eight, this was the only reoperation. Five patients required six reoperations due other reasons including colonic ischemia, severe peripheral vascular disease leading to limb ischemia, suspected small bowel ischemia (no resection was required), and ischemic omentum. Of note, none of the patients that were reoperated underwent a preceding interventional radiology procedure. Nevertheless, six patients that were reoperated required additional interventional radiology procedures after the second surgery. This included a percutaneous drainage of an intraabdominal collection in four patients, PTD placement in one patient, and angioembolization of a GDA stump pseudoaneurysm in another.

Among patients that were not reoperated, 10 underwent percutaneous drainage of an intra-abdominal abscess, 3 underwent angioembolization of pseudoaneurysm, and another patient underwent an insertion of a dilating stent to the celiac trunk due to severe stenosis.

Demographics and comorbidities of patients that underwent reoperation as compared to those who did not, are described in Table 2. No differences in age, gender, comorbidities, or serum levels of tumor markers were found between groups. Patients that required reoperation had significantly higher preoperative plasma bilirubin levels (7.3 ± 6 vs 4.19 ± 5.4; *P* = 0.048, Table 2). No significant differences were found in the rate of preoperative endoscopic retrograde cholangiopancreatography (ERCP) and stent placement between groups (39.1% vs 25.9% in cancer patients that underwent reoperation compared to those that did not; *P* = 0.259, Table 2).

**Table 2:** Patient characteristics and perioperative data

	Reoperation *n* = 48	No reoperation *n* = 385	*P*
Gender, m:f (ratio)	27:21 (1.28)	217:168 (1.29)	0.88
Age (years)	66.5 ± 11.4	64.6 ± 11.9	0.3
Comorbidities:			
HTN, *n* (%)	14 (29.1)	97 (25.1)	0.57
IHD, *n* (%)	5 (10.4)	37 (9.6)	0.64
DM, *n* (%)	9 (18.7)	118 (30.6)	0.19
COPD, *n* (%)	1 (2)	2 (0.5)	< 0.001
Pre-op Hb	12.64 ± 1.5	12.27 ± 1.6	0.18
CEA	3.73 ± 6.0	5.52 ± 14.8	0.54
CA19-9	174 ± 289	209 ± 309	0.57
Pre-op bilirubin	7.3 ± 6	4.19 ± 5.4	0.048
Pre-op GGT	523 ± 497	458 ± 559	0.53
Neo-adjuvant Tx, *n* (%)	2 (4.1)	12 (3.11)	0.14
Benign pathology, *n* (%)	2 (4.1)	31 (8)	0.13
Malignant pathology, *n* (%)	46 (95.9)	354 (91.9)	0.013
PDAC, *n* (%)	28 (58.3)	198 (51.4)	0.98
Ampullary CA, *n* (%)	6 (12.5)	47 (12.2)	0.814
Duodenal CA, *n* (%)	1 (2)	15 (3.8)	0.014
CCA, *n* (%)	5 (10.4)	15 (3.9)	< 0.001
ERCP + stenting (all malignant), *n* (%)	18 (39.1)	92 (25.9)	0.259
ERCP + stenting (of CA, not CCA), *n* (%)	12 (34.2)	67 (25.5)	0.57
ERCP + stenting (of CCA), *n* (%)	2 (40)	11 (73.3)	0.15
Staging of CA patients			
T1, *n* (%)	11 (27.5)	90 (32.4)	0.816
T2, *n* (%)	13 (32.5)	108 (38.9)	0.85
T3, *n* (%)	6 (15)	31 (11.2)	0.23
T4, *n* (%)	10 (25)	48 (17.4)	0.2
At least one positive LN, *n* (%)	20 (50)	140 (50.9)	0.87
No. of positive LN, average (range)	2.3 (1–7)	3.2 (1–21)	0.19
NET, *n* (%)	2 (4.16)	31 (8)	0.01
IPMN, *n* (%)	1 (2)	40 (10.3)	< 0.001
Readmission, *n* (%)	11 (22.9)	66 (17.1)	0.24
DGE, *n* (%)	3 (6.2)	57 (14.8)	0.002
Wound infection, *n* (%)	4 (8.3)	63 (16.3)	0.019
B/C pancreatic fistula, *n* (%)	7 (14.5)	57 (14.8)	0.93
Tracheostomy, *n* (%)	7 (14.5)	2 (0.52)	< 0.001
LOS (days), average (range)	39.07 ± 30.15	16.5 ± 10.3	< 0.001
60 days’ mortality, *n* (%)	9 (18.75)	10 (2.6)	< 0.001
Adjuvant Tx, *n* (% of CA patients)	23 (57.5)	181 (65.8)	0.58

*HTN* hypertension, *IHD* ischemic heart disease, *DM* diabetes mellitus, *COPD* chronic obstructive pulmonary disease, *Ca* carcinoma, *CEA* carcinoembryonic antigen, *GGT* gamma glutamyl transferase, *Tx* treatment, *PDAC* pancreatic adenocarcinoma, *CCA* cholangiocarcinoma, *ERCP* endoscopic retrograde cholangiopancreatography, *LN* lymph node, *NET* neuroendocrine tumor, *IPMN* intraductal papillary mucinous neoplasm, *DGE* delayed gastric emptying. *LOS* length of stay

Patients with CCA had the highest rate of reoperations (25%), followed by PDAC (12.4%), ampullary carcinoma (11.3%), NET (4.16%), and IPMN (2%) (Table 2). Of note, no significant differences in the rates of preoperative ERCP and stent placement were demonstrated between patients with CCA and those with non CCA carcinoma, and ERCP was not associated with increased rate of reoperation in neither (Table 2). No significant differences in TNM staging were demonstrated between patients with PDAC that underwent reoperation and those who did not (Table 2). No difference in the rate of patients that received neo-adjuvant therapy was demonstrated between the groups (Table 2). The readmission rates and rates of level B or C POPF were comparable between groups (Table 2).

Patients that required reoperation had significantly longer LOS compared to those that did not (an average of 39 vs 17 days), and 14.5% required prolonged mechanical ventilation and tracheostomy, compared to only 0.5% (*P* < 0.001). Most remarkably, their 60-day mortality rate was dramatically increased to over 7-fold (18.3% compared to 2.6% in those that did not have reoperation). Six patients underwent more than one reoperation. Four had two reoperations and two patients had four and six. Indications for these surgeries included anastomotic leak, bleeding, wound dehiscence, sepsis, and second and third explorations for suspected mesenteric ischemia. Patients that underwent a single reoperation had a 14.2% mortality rate, compared to 50% in those who underwent more than one reoperation. All patients that underwent more than two reoperations died during the post-operative course. No significant differences in age, gender, demographics, or the characteristics primary disease were noticed between patients that died following a reoperation and those who survived.

**Fig. 1:**
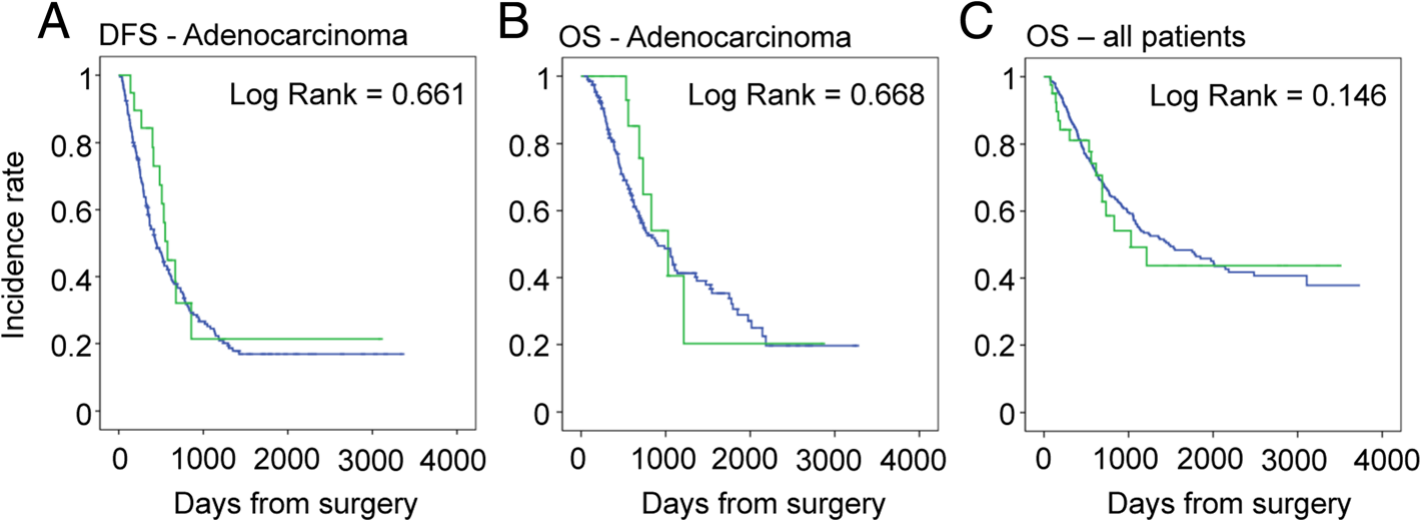
Long-term outcomes of reoperation following PD. **a** Disease-free survival and **b** overall survival of patients with adenocarcinoma with (green) and without (blue) a reoperation. **c** Overall survival of patients that underwent PD with (green) and without (blue) a reoperation

The rate of completion of adjuvant chemotherapy was comparable between patients with cancer (PDAC, CCA, Ampullary CA, and Duodenal CA combined) that underwent reoperation, and those who did not (57.5% vs 65.8% respectively; *P* = 0.58). In PDAC patients, reoperation had no impact on long-term oncologic outcome. The median disease-free survival (DFS) of PDAC patients that underwent a reoperation was 576 ± 45 days vs 446 ± 56 days in those who underwent a reoperation (*P =* 0.416). The overall survival (OS) of PDAC patients was 907 ± 130 vs 1029 ± 202 days (*P =* 0.416). Finally, taking all patients into account, there was no difference in OS between those who underwent a reoperation and those who did not (Fig. 1).

## Discussion

PD is the only curative option for pancreatic head, duodenal and distal common bile duct malignancy. High volume centers for pancreatic surgery perform this procedure routinely with acceptable morbidity and mortality. Nevertheless, the overall post-operative complication rate of PD remains relatively high, compared to other abdominal procedures, even in specialized centers [2, 4, 14, 15].

Multiple factors lead to increased complexity of perioperative management of PD patients in recent years, including older age, higher rates of patient with borderline resectable tumor undergoing PD with vascular resections, and more frequent use of neoadjuvant chemotherapy. One of the surrogate markers of poor surgical outcome of any procedure is the need for reoperation in the post-operative period. In this study, we assessed the indications for early reoperation following PD and its short- and long-term consequences.

A little over 10% of patients suffered from post-operative complications that required at least one reoperation in the post-operative course. The majority were performed due to anastomotic leaks and PPH, followed by wound dehiscence and evisceration. These indications were also demonstrated in other large series as leading causes of reoperation [1–3, 7, 11, 13]. With the growing use of invasive radiology for controlling delayed PPH, one could estimate that the rates of reoperation due to bleeding will significantly decrease. This may be true for patient with delayed PPH that are hemodynamically stable and in which pseudoaneurysm of the GDA is the most likely source, such as in cases of long standing pancreatic fistula.

Nevertheless, in early PPH, in which the source of bleeding is uncertain, and the patient is unstable, it is our opinion that the role of IR is still limited, and OR is the appropriate option. The majority of our cases were of the later kind. Other indications, including bowel ischemia and sepsis of unknown origin, were also common [1–3]. Although infrequent, a leak from GJ is known to have dire consequences [1–3], and indeed this was the most common cause of reoperation among all anastomoses, despite higher frequency of PJ and HJ leaks, as most could be managed conservatively.

Drains were left in all of our patients. Many studies have assessed the benefits and drawbacks of routine drain placement during PD. Of these, several have demonstrated that omission of drains did not result in higher rates of post-operative complications, and even reduced the rates of clinically relevant pancreatic fistula and fistula-associated complications [16, 17]. On the other hand, other high-quality studies showed significantly increased rates of post-operative complications and mortality when drains were not routinely used [18]. In light of this ongoing controversy, we still maintain our tradition to routinely place drains during PD. It is beyond the scope of this study to assess the effect drains on timing and outcomes of reoperation.

The vast majority of patients underwent PD for malignant diseases. Among them, there was a significantly high rate of CCA patients that required reoperation (25%). A possible explanation for this might have been an increased rate of preoperative biliary drainage and stent placement in this patient subgroup, resulting in preoperative contamination of the biliary systems with a foreign body. However, in our cohort, the rate of preoperative biliary drainage and stent placement in CCA patients was comparable with that of other malignancies [19, 20].

As expected, the length of stay of reoperated patients was significantly longer (more than doubled on average), and over 14% required tracheostomy for prolonged mechanical ventilation and ICU stay, comparing to only 0.5% of those that were not reoperated. The overall toll of a reoperation is reflected by the substantially increased 60-day mortality rate, over 7-fold higher in patients that required reoperation, with risk of death rising proportionally to the number of reoperations. Somewhat surprisingly, when ultimately discharged, patients that underwent reoperation had comparable rates of readmission to those who had uneventful post-operative course. This trend was also shown by previous studies of reoperation following PD [11, 13].

It has long been suggested that post-operative complications may affect the long-term outcome of oncologic patients, both directly, by influencing host immunity and by preventing patients from receiving adjuvant treatment [21, 22]. In colorectal cancer, for example, large series have shown that anastomotic leaks are associated with shorter DFS and decreased OS [22–26]. One possible explanation is that post-operative complications lead to sustained surge of both local and systemic inflammatory mediators, which may play a role in tumor progression [27–30]. On the other hand, other studies contradict this observation, by showing no significant impact of anastomotic leaks or other major complications on disease recurrence and survival [31–34]. In pancreatic cancer, this controversy remains, as some demonstrated worse long-term oncologic outcome in patients who developed POPF and other post-operative complications [35–37], while others showed no long-term effect. Nevertheless, it should be remembered that when dealing with pancreatic cancer, aside from the local and systemic effects of post-operative complications, the prolonged length of hospital stay may delay administration of adjuvant therapy and further worsen the already poor prognosis [36, 38]. Cho et al. showed that the presence of major post-operative complications following PD is an independent prognostic factor for poor survival and distant recurrence in periampullary cancer patients [38]. In our study, major post-operative complications requiring reoperations in PDAC patients, was not associated with decreased rates of adjuvant chemotherapy administration and somewhat surprisingly, their long-term OS was comparable to those who did not undergo reoperation. This lack of significant difference between the groups, even if attributed to the relatively small cohort of reoperated patients, highlights the poor general long-term outcome of pancreatic cancer, with or without a reoperation.

It has always been our belief that patients undergoing PD have somewhat limited reserve and ability to withstand a “second hit,” such as massive bleeding or sepsis. Therefore, whenever a patient was stable, we always deferred to conservative means, mostly by IR. Nonetheless, whenever patients had signs of severe shock, partial or no fast response to treatment, or signs of generalized peritonitis, we took them back to the OR for source control, be it bleeding or leak. Reoperation may indeed carry a major toll, as reflected in our study, and we cannot tell that this manner is not responsible for at least part of the grave outcome in the reoperated cohort. Nonetheless, it is very difficult to assess what would have been the outcome of our patients, if we would not have operated on them.

The overall mortality of our patients is not different from that reported by many other tertiary centers, and not increasing over time, even though we now operate on older patients and added vascular resections in the last 5 years of the study.

In conclusion, this study shed light on the devastating impact of severe post-operative complications requiring reoperation in patients undergoing PD.

The need to reoperate indicated a group of patients that would require particularly long hospital stay, often in the ICU, with prolonged mechanical ventilation and tracheostomy, and most strikingly, that had an extremely high mortality rate of over 18%. Nevertheless, we could not demonstrate an effect on long-term oncologic outcome, an observation that may reflect the aggressive nature of PDAC.
